# Planning the Future Oral Health Workforce: A Rapid Review of Supply, Demand and Need Models, Data Sources and Skill Mix Considerations

**DOI:** 10.3390/ijerph18062891

**Published:** 2021-03-12

**Authors:** Madhan Balasubramanian, Aliya Hasan, Suruchi Ganbavale, Anfal Alolayah, Jennifer Gallagher

**Affiliations:** 1 Faculty of Medicine and Health, The University of Sydney, Sydney 2006, Australia; 2 Australian Research Centre for Population Oral Health, Adelaide Dental School, The University of Adelaide, Adelaide 5005, Australia; 3 Faculty of Dentistry, Oral & Craniofacial Sciences, Kings College London, London SE5 9RS, UK; aliya.hasan@kcl.ac.uk (A.H.); SURUCHI.Ganbavale5@myport.ac.uk (S.G.); anfal.alolayah@kcl.ac.uk (A.A.); jenny.gallagher@kcl.ac.uk (J.G.); 4 Dental Academy, William Beatty Building, Hampshire Terrace, University of Portsmouth, Portsmouth PO1 2QG, UK

**Keywords:** health workforce, operational models, planning, skill mix, integration

## Abstract

Over the last decade, there has been a renewed interest in oral health workforce planning. The purpose of this review is to examine oral health workforce planning models on supply, demand and needs, mainly in respect to their data sources, modelling technique and use of skill mix. A limited search was carried out on PubMed and Web of Science for published scientific articles on oral health workforce planning models between 2010 to 2020. No restrictions were placed on the type of modelling philosophy, and all studies including supply, demand or needs based models were included. Rapid review methods guided the review process. Twenty-three studies from 15 countries were included in the review. A majority were from high-income countries (*n* = 17). Dentists were the sole oral health workforce group modelled in 13 studies; only five studies included skill mix (allied dental personnel) considerations. The most common application of modelling was a workforce to population ratio or a needs-based demand weighted variant. Nearly all studies presented weaknesses in modelling process due to the limitations in data sources and/or non-availability of the necessary data to inform oral health workforce planning. Skill mix considerations in planning models were also limited to horizontal integration within oral health professionals. Planning for the future oral health workforce is heavily reliant on quality data being available for supply, demand and needs models. Integrated methodologies that expand skill mix considerations and account for uncertainty are essential for future planning exercises.

## 1. Introduction

The health workforce is the backbone of health systems, fundamental towards achieving universal health coverage (UHC) and meeting sustainable development goals (SDGs) [1,2,3]. Planning for the future health workforce is a complex process, requiring trade-offs across multiple health professional objectives in education, training and regulation, and numerous uncertainties due to transition health environments (demographic, epidemiologic and technology) [4]. In general health workforce planning aims to achieve a proper balance between supply and demand of health professionals [5]. The philosophy behind planning is to ensure the right number of health personnel, with the right training and skill sets are available at the right place and at the right time to meet population needs, but at an acceptable cost and quality [6]. The process is not just technical, but a political one [6]. Planning decisions on the number, type and distribution of health personnel depend and are influenced by a range of social, economic and professional values enshrined within underlying health systems. 

Planning for the future oral health workforce presents its unique challenges. First, the profession of dentistry, in many countries, has remained historically ‘distinct’ from the medical, nursing and broader health professions [7]. Silos are visible in the education and practice of dental professionals, that also extend to policy and planning decisions [7,8,9]. Second, dentists are at the center of the dental profession, entrusted with the responsibility of providing leadership, and serving as the first point of contact for the majority of oral health conditions [7]. The allied dental workforce—dental hygienists, dental therapists, dual qualified hygienist/therapists, dental technicians, denturists, prosthetists and dental assistants—support the dentist in the provision of care. However, the acceptance of allied dental professionals vary country to country and potentially reflect on the use of skill mix in planning decisions [10]. Third, dental specialists are the gatekeepers of the profession, providing exceptional services to ‘special’ patients, and serving as a focal point for quality benchmarks, innovation and adoption of new procedures, clinical research and education of the dental team [11]. Atleast 10 distinct specialist dental professionals exist: orthodontists, oral and maxillofacial surgeons, prosthodontists, periodontists, endodontists, paediatric dentists, oral pathologists, oral medicine, special needs and dental public health specialists. Not all dental specialities gain equal importance in the planning exercise. A further challenge in oral health workforce planning is consideration for both horizontal (i.e., within profession skill mix) and vertical (i.e., skill mix outside the dental profession) integration in planning models.

Traditionally, four broad approaches to health workforce planning have been identified in the literature: needs-based, utilisation or demand-based, health workforce to population ratio, and target setting approach [4,12]. Each of these approaches includes atleast one or more of the basic building blocks in modelling: supply, demand and need [13]. Supply models estimate the number of health personnel available based on the current stocks, flows/migration, and newly trained personnel. Demand or needs model estimate health personnel required to meet the underlying population demand or needs respectively. Needs are identified through epidemiological surveys, accounting for diseases prevalence and health status. Demand is identified through health service utilisation. Supply and demand/needs models are usually presented together, so the combined model can determine the gap in health personnel availability. Planning models are also classified as being deterministic or stochastic [12]. Deterministic models assume the outcome is certain, and always deliver the same results for the same input values. On the other hand, stochastic models allow for the introduction of random changes and provide means for building an element of uncertainty in the overall planning models.

Over the last decade, there has been a renewed interest in oral health workforce planning [14,15,16]. The purpose of this review is to examine oral health workforce planning models on supply, demand and needs, mainly in respect to their data sources, modelling technique and use of skill mix. We also identify strengths and weaknesses in these workforce models and provide insights on how oral health workforce planning can evolve in the future to meet changing population needs and demands, improving health outcomes and health systems performance.

## 2. Methods

The study was based on a rapid review approach adapted from Khangura et al. [17] and Thomas et al. [18]. Rapid reviews are a type of systematic reviews, where components of a regular systematic review are simplified or made more efficient to produce information in a shorter span of time, but with minimal impact to quality [19]. In recent years, rapid reviews have emerged as an efficient solution to synthesizing evidence to support health policymaking and health systems strengthening by providing high-quality evidence in a timely and cost-effective manner [20]. Our rapid review involved the following steps: (i) defining a review/research question (ii) developing a search strategy (iii) establishing selection/eligibility criteria (iv) screening and study selection (v) data extraction and (vi) synthesis of findings. We have adhered to Khangura et al.’s descriptive synthesis of findings and emphasis on translation of findings to policy and practice [18,19]. Targeted searching of key databases, and data abstraction by mapping study characteristics were adopted from Thomas et al. [17,19]. While no generic trend or adherence to any particular variant of rapid reviews have been observed in recent reviews [19,21], our methodological underpinning to key schools of rapid review thought streamlines our approach and philosophy.

### 2.1. Research Question

The following question was formulated for this part of the review: What are the main operational models, data sources and techniques used in oral health workforce planning?

### 2.2. Search Strategy

A comprehensive search strategy was designed in consultation with an expert librarian at Kings College London to capture the relevant literature on the topic of interest. We included four broad categories in our search criteria: healthcare and workforce planning, dental service provision, dental staffing, modelling techniques and skill mix. Specific MeSH terms and keywords, along with Boolean operators were used to build the search. This list was refined by conducting a group discussion among all authors to arrive at a consensus. Electronic searches were carried out in 2 different databases: PubMed and Web of Science. The search strategy was designed for PubMed interface, and later revised for Web of Science. A limited literature search was undertaken for relevant titles, abstracts and keywords (please see Supplementary Tables S1 and S4). Standard techniques such as using truncation methods and searching for relevant references from the bibliography provided in searched papers and were also used. Manual forward-backward search or citation tracking of the identified articles were performed using Scopus and Google Scholar. The search process and identification of articles was carried in the second half of 2020, between September and December.

### 2.3. Eligibility Criteria

Published original research articles on oral health workforce planning were included in the review. Studies need to have followed a workforce modelling approach to estimate the current or future requirements of oral health personnel (dentists, dental specialists, therapists, hygienists, or other allied dental personnel). No restrictions were placed on the type of modelling philosophy, and all studies including supply, demand or needs-based estimates were included. Studies could range from simple dentist population ratios, to more complex skill mix and scenario-based models. Articles published between 2010 and 2020 in English language were included. 

Commentaries, reviews, policy briefs, government reports, working papers, opinions, perspectives, conference abstracts, letter to editors, dissertations/thesis, or evidence summaries were excluded in this review. Oral health workforce modelling should have been the main aspect of the paper—studies that only identified oral health workforce requirements without any supporting methods or modelling approaches were excluded. Studies should have also focused on oral health personnel as the basic unit for modelling—studies focusing on dental practices or facilities were excluded.

### 2.4. Study Selection

First, one of the reviewers (S.G.) identified all articles via database searching, duplicates were excluded and imported the final list into a web/mobile based systematic review management application called Rayyan (Qatar Computing Research Institute, Doha, Qatar) [22]. The tool is mainly designed to expedite the initial screening of abstracts, titles and keywords using a process of semi-automation while incorporating a high level of usability [22]. Duplicates were removed. Four reviewers (M.B., A.H., A.A., S.G.) carried out the selection of articles. Articles that did not fit the eligibility criteria were excluded. If limited information was available in the initial scanning process, the full text was obtained to determine eligibility. Later, the full text of all selected articles was read, and further limited to only relevant articles based on the selection criteria. Lack of agreement or conflict arising in the selection of articles were resolved through group discussions and consultation with the senior author (J.G.).

### 2.5. Data Extraction, Synthesis and Reporting

Extraction of data from selected papers was performed by using pre-defined criteria. We extracted a range of study characteristics including: author/year, country of research, aim of study, workforce/population modelled, model type, supply/demand/needs models, data sources, findings, strengths/limitations, policy implications and conclusion. All authors were involved in discussing the emerging data to decide on relevance and decide any modifications in the data extraction framework for the study. Data extraction was conducted using an MS Excel template, which was later developed into a MS Access database for improved usability. We followed a descriptive approach in synthesis and reporting of data, based on Khangura et al.’s rapid review methodology [18]. The focus of this paper is limited to detailed characteristics of the supply, demand and needs model, how these models were developed and the sources of data for these models.

## 3. Results

**Figure 1:**
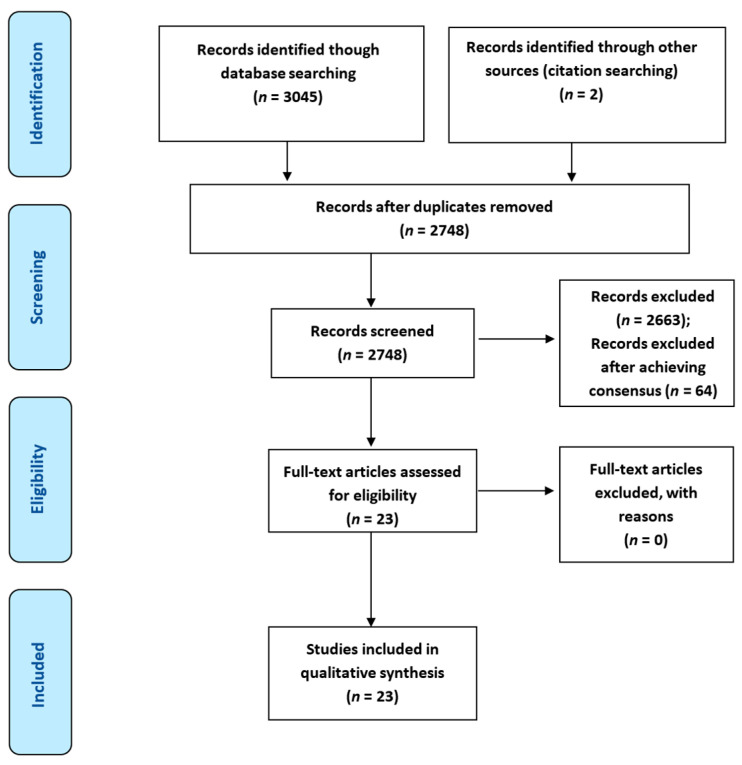
PRISMA Flowchart for the Rapid Review.

A total of *n* = 3047 potential articles were identified through database and citation searching. Following the removal of duplicates, *n* = 2748 articles were available for title/abstract/keyword screening. A total of *n* = 2727 articles were excluded (*n* = 64 after group discussion and conflict resolution), providing *n* = 23 articles for data extraction and qualitative synthesis. Figure 1 provides the PRISMA flowchart of the study selection. A list of selected studies for the rapid review will full citation of articles is provided in Supplementary Table S3.

### 3.1. Main Study Characteristics

The main characteristics of the 23 selected studies are provided in Table 1. These publications were from 15 different countries across the world: Australia [23], Canada [24], Chile [25], China [26,27], Japan [28], Kuwait [29], India [30], Ireland [31], Malaysia [32,33], Oman [34], Sri Lanka [35], Taiwan [36], Trinidad & Tobago [37], the United Kingdom [38,39,40,41], and the United States of America [42,43,44,45]. Seven studies were based in the WHO American Region, followed by the European (*n* = 5) and Western Pacific Regions (*n* = 4). Most of the studies were also based on high-income group World Bank countries (*n* = 17). It is important to note that no studies were identified from the WHO African Region or low-income group World Bank countries Dentists were the dominant oral health workforce group modelled across 13 studies [24,25,26,27,28,29,30,31,34,35,36,43,44]. Five studies considered both dental and allied dental workforce (including therapists, hygienists, clinical technicians, denturists) in the workforce models [32,33,38,39,40]. Four studies specifically modelled the dental specialist workforce, including all dental specialities [41] or covering any of the limited specialist groups: oral and maxillofacial surgeons [23], orthodontists [37] or pediatric dentists [45]. One study has modelled all three oral health workforce groups: dentists, pediatric dentists (specialists) and dental hygienists (allied dental professionals) [42]. The population being modelled in the studies ranged from the full population of the country/region (*n* = 10) [23,25,28,29,30,34,35,36,40,43] or limited to include a specific group such as children (*n* = 4) [26,37,42,45], adults (*n* = 3) [31,32,33], or older people (*n* = 1) [38]. Four studies focused on population-based at a specific catchment area such as province/state (Liaoning Province, China [27]; Kentucky, USA [44]; Georgia, USA [42]) or a service/administrative zone (South Central Strategic Health Authority, England/UK [39]; Canadian Armed Forces service areas, Canada [24])

A number of workforce modelling types were observed in the selected studies, with the most common application being the workforce to population ratio (*n* = 10) [25,27,28,29,30,34,36,37,41,44] followed by a needs-based/demand-weighted (*n* = 5) [23,35,38,39,45] variant. One article compared both the workforce to population ratio and needs based demand weighted models in the same study [24]. Four studies used a needs-based model [26,31,32,33]; and three a demand or utilization based model alone [40,42,43]. 

### 3.2. Detailed Study Characteristics

Table 2 presents detailed study characteristics of the supply, demand and needs models along with various data sources and techniques used in developing these models.

#### 3.2.1. Supply Models and Data Sources

A total of 18 studies in the review have presented supply models. Existing stock of the dental workforce has been determined in all these studies, with the most common estimation being through the use of dentist registrations data, obtained via a national dental council or a regulatory authority (*n* = 7) [29,30,31,35,38,41,42,44]. Two studies from the USA have determined estimates using state dental regulatory authorities, namely from Georgia [42] and Kentucky [44]. Brailsford & De Silva [35], prepared a separate national register for the study accommodating registrations, record matching and panel interview to identify existing stock and currency of practice. Gallagher et al. [38] used a range of sources (registrations, dental practice survey and NHS government data) in determining the existing stock of oral health workforce in England, UK. In addition, four other studies have used mainly dentist surveys in accounting for existing workforce numbers [23,27,28,43]. Studies in Australia [23] and Japan [28] have utilized national dental workforce surveys in determining more detailed estimates on the stock of dentists. A few studies have also used government data from sector specific areas such as health services [36,38,39,42] or armed forces [24]. 

Surdu et al. [45] documented an elaborate use of national dental association registrations data for determining supply estimates of pediatric dentists, in addition to survey and workforce publications from government sources. Nine studies have included flow estimates within their supply models, through the inclusion of migration, retirement, absence, return to work and deaths [23,28,31,34,35,36,38,39,45]. Ten studies have included newly trained dentists in the supply model [23,25,28,30,31,34,35,36,38,39,45], mostly through information available from dental school completions. Brailsford & De Silva [35] also incorporated a student survey to understand student motivations and career expectations. A few studies have also accommodated government regulations and potential for newly created dentists/hygienist places in their supply estimates [34,38,39].

Studies have represented overall workforce participation either through dental personnel numbers alone (*n* = 5) [25,29,30,34,44] or accounting for clinical or part time hours worked and determining full time equivalent dentists (*n* = 7) [24,31,35,38,39,42,45]. Ju et al. [23] and Ishimaru et al. [28] have used work status questions from surveys in determining workforce participation.

#### 3.2.2. Demand Models, Population Only Estimates and Data Sources

Demand models, represented as a needs-based demand weighted or utilization/demand model were presented in nine studies [23,35,38,39,40,42,43,45]. At the basic level estimates were presented as only population numbers in eight studies [25,27,28,29,30,34,36,44]. Population estimates were sourced from national or state-based census sources, government departments, or a combination of both. Seven studies [23,24,35,38,39,40,45] estimated the expressed demand through available data on oral health status, and converted the demand to workforce requirements as minutes, dentists or FTE dentists. Brailsford De Silva [35] used FDI/WHO method in estimating services needed per person—based on people who actively express the need for care from a population survey. Three studies in the UK (Gallagher [38,39]; Wanyoyi, [40]) have used NHS treatment data to arrive at a very detailed estimates of demand and workforce requirement. A simple estimation of demand was reported in Eklund and Balit [43]—the proportion of dental visits people make in a year (determined form a previous publication) in estimating workforce requirements.

#### 3.2.3. Needs Models and Data Sources

The review identified four studies [26,31,32,33] that have predominantly used a needs model in determining workforce requirements. All four studies used a population survey to determine oral health status and treatment needs. Three studies were limited in survey design or sample size or research question: Sun et al. [26] surveyed only 12-year-olds in China, and Ab-Murat et al. [32,33] surveyed 30–54-year-old university employees at a single site (public university) in Malaysia. Ab-Murat et al. [32,33] also focused on specific aspects covering periodontal and prosthodontic treatment needs, which were measured through two approaches: a normative approach and socio dental approach. Face-to-face questionnaires were also used to determine oral health impacts and behaviours. Both Ab-Murat et al. [32,33] and Sun et al. [26] used panel interviews to determine treatment timings, helping in the estimation of workforce requirement. Sun et al. [26] further expanded the needs aspect (determined for 12-year olds) to whole population in China by utilizing care provision ratios, adopted from a previous study. In contrast, Ahern et al. (2019) [31] used a more comprehensive oral health survey dataset that covered all adults (15+ years old) in Ireland. The population survey included questions on oral health status, behaviours, impacts and visiting patterns to determine service timings and workforce requirements in FTE dentists.

#### 3.2.4. Skill Mix Considerations

The use of skill mix in modelling that take into account the contribution or influence of different workforce groups towards supply, demand and/or need models has been limited. Only seven studies accounted for skill mix variations [32,33,38,39,40,42,45]. The common application of skill mix was the use of allied dental teams (dental therapists, hygienists, denturists) along with dentists [32,33,38,39,40,42]. Surdu et al. [45] have applied specialist pediatric dentists along with general dentists in skill mix models for planning pediatric dental workforce. Studies that used skill mix accounted for changes in the provision of services by the extended dental team and how their participation effectively altered the future workforce requirements for oral health care. None of the studies examined the provision of oral health care outside the main oral health workforce groups i.e., accounting for possible care provision by medical, nursing, pharmacy or broader allied health workforce teams. While a few studies have discussed the concept of skill mix within dental teams, they haven’t included it within the modelling approaches.

Almost all the models being presented were deterministic; only one study included a stochastic element in their modelling approach [38]. A number of studies identified limitations in relation to data sources, either data being unavailable or on the quality of planning data. Other limitations highlighted were being single site studies, small sample size (see Supplementary Table S3).

## 4. Discussion

The review examined oral health workforce planning models within the published scientific literature over the last 10 years. Many studies were from high-income countries; no studies were identified from low-income countries and the WHO African region. Calculating workforce to population ratios were the most common modelling approach, followed by needs-based demand weighted approaches. Needs-based approaches had limitations in the population being studied and/or the nature of oral health need assessments being undertaken. Lack of quality data for the modelling exercise is omnipresent in all sources of supply, demand and needs. Very few studies have made use of skill mix considerations in their models. Studies have not accounted for uncertainty of outcomes, or randomness in their modelling exercises, and were mostly deterministic in nature.

Workforce to population ratios, though commonly used in oral health workforce planning studies, represent a crude ratio and bring several shortcomings to the planning process. First, this ratio is based on assumptions of homogeneity across the numerator (i.e., all dental personnel are active and equally productive and will remain so) and that the denominator (i.e., all populations) will have similar oral health needs and will remain constant) [6,12]. This ratio does less justice to address differences in dentist practice activity or productivity (across age, sex, levels of experience, area of practice) or varying levels of oral disease prevalence, dental care utilisation or demographic, socio-economic differences of across population groups. Second, maldistribution of health personnel across different geographic areas, practice types (public or private) or facilities (hospitals, clinics) cannot be adequately represented using a single workforce to population ratio [5]. While it is possible to offer some comparisons using workforce to population ratios at global, region, country, state/area, facility levels, its inability to account for the intrinsic differences in dentist and disease characteristics would still prevail. Third, the ratio does not help us in understanding progress made in achieving wider health system objectives and performance benchmarks in regard to accessibility, equity, quality and efficiency, [6] particularly as the most basic aspect of access ‘coverage’ within countries can differ, particularly between urban and rural areas (ref)Nevertheless, the workforce to population ratio approach is less demanding in terms of data and it brings simplicity in terms of providing a snapshot estimate to health planners [4,5,46]. Our review identified studies from Kuwait [29], Trinidad and Tobago [37], Chile [25], Oman [34], Taiwan [36], India [30], China [26], UK (dental specialists) [41], and Kentucky (USA) [44] using a workforce to population ratio approach. It should be noted that all these studies also identified limitations in data sources or non-availability of quality data and they have resorted to using workforce to population ratio as a means of commencing the oral health workforce planning process [47].

Demand based planning approaches primarily make use of health service utilisation data. Our review has identified studies that use both dentist surveys [43] and administrative data such as electronic health records (EHR) [40] for extracting oral health service utilisation data. Traditionally, survey-based methods have been popular in understanding practice activity of oral health personnel, and the nature and type of services they offer to patients. For example, in Australia, dentist practice activity surveys have been the cornerstone of oral health workforce policy and planning since early 1980′s [48,49,50,51]. In recent years, however, the adoption and use of computerised systems and use of EHRs in dental practices and hospitals are becoming more common in many counties [52]. EHRs provide a viable, cost efficient and timely alternative to understand dental service utilisation data, against surveys that are more time consuming and resource intensive [53,54]. However, the use of EHRs is still in its infancy in terms of data quality and consistency in systems across public and private sectors [55,56,57]. As a large proportion of dentists practice in the private sector [7], it becomes important to find avenues to improve consistency as well as building data repositories for research and planning purposes. The International Association for Dental Research (the peak global dental research body), and its Network for Practice Based Research [58] has raised the importance of partnerships across private and public dental sector and Universities to improve quality, consistency and use of oral health service data collected in dental clinics or hospitals for research purposes.

Needs based approaches are more reflective of the underlying oral health needs of population. Such models take into account oral health conditions such as caries levels, periodontal status or missing teeth [26,31,32,33]. Data for needs in selected studies in the review are from population oral health surveys. However, not all studies were comprehensive, and were limited in the type of population group being surveyed or type of needs being assessed, or oral health status questions being studied.

The use of skill mix is a vital component in health workforce models, as it helps to accommodate task sharing and team work both across members of the dental team and wider medical, nursing and allied health teams. Prior planning theories and methods have to failed to incorporate skill-mix in planning designs [59]. The use of skill mix is an important factor when undergoing oral health workforce planning as it helps to determine the future framework of the oral health team in terms of number, size and consequently patient base [38]. In order to predict the future of the oral health workforce, it is important to appreciate the changes within society in terms of comparing and contrasting oral health need and demands, whilst balancing this against the supply of the dental workforce [60]. Vertical integration of the oral health workforce with other health professionals is also vital moving in the future, as in a post COVID era its logical to argue for greater collaboration with all members of the medical, dental and social teams so as to meet the growing needs and demands of the population [7]. Future research in oral health workforce planning needs to accommodate both horizontal and vertical integration within their planning exercises. A major issue for concern is planning the future dental speciality workforce. As gatekeepers of the dental profession, dental specialists are vital towards setting quality benchmarks, identifying divers for innovation and change. Planning exercises will need to extend to involve dental specialists along with general dentists and other members of the dental team in order to best serve the needs of the population.

The study identified lack of consistency and quality of workforce data arising from a wide variety of data sources. Supply data sources have particularly problematic due to the number of sources required to identify the stock, flows and newly trained. Our prior research has identified a range of inconsistencies across countries in these supply data sources and necessity for advocacy and solutions in improving registration and migration data on dental professionals [8].

### Limitations

This rapid review included only published scientific research articles between 2010 to 2020. We limited our focus only on the past decade, as several advocacy and major progress on health workforce planning by global, regional and national organisations were prevalent during this period. Health workforce planning could also be conducted as an ‘in-house’ exercise by planning organisations which is more prevalent in grey literature. Our decision to include only published peer reviewed articles was conscious, mainly due to the rapid review method adopted [17,18], but also able to understand how well oral health workforce planning is represented in our scientific literature. One possible limitation is the fact that the senior author of this review is also active in workforce modelling and has authored several of the publications; however, we took account of this by having a wider research team work on the data extraction and analysis, recognising the importance also of having an expert in the field involved. We also identify a recent review published by a different group of colleagues on a similar topic [61]. Whilst our study differs in terms of review question, methods, and framework used in synthesis, the findings together make a major contribution to this important but relatively unexplored field of oral health workforce planning. The purpose of our review was to distinctly focus on data sources, techniques and skill mix considerations. Our method of synthesis was comprehensive to the above three parameters.

## 5. Conclusions

Planning for the future oral health workforce is heavily reliant on quality data being available for supply, demand and needs models. Studies have presented with a lack of uniformity and accepted standards in oral health workforce modelling approaches and reporting. Integrated methodologies that expand the skill mix considerations and introduce randomness and system dynamics to account for uncertainty are essential for future planning exercises.

## Figures and Tables

**Table 1:** Main characteristics of selected studies in the rapid review.

Study No.	Author(s), Year	Aim of Study	Country; WHO Region; World Bank (WB) Group	Workforce Group Modelled	Population Modelled	Model Type
1	Ab-Murat N et al., 2015	To compare estimates of periodontal dental treatment needs and workforce requirements for different skill mix models using normative and sociodental approaches	Malaysia; WHO South East Asian Region; WB Upper Middle Income Group	Dentists and Dental Therapists	Malaysian adults; 30–54 years old	Needs-based
2	Ab-Murat N et al., 2015	To estimate and compare prosthodontic treatment needs and workforce requirements, using the normative and the sociodental approaches for different skill mix models	Malaysia; WHO South East Asian Region; WB Upper Middle Income Group	Dentists and Denturists	Malaysian adults; 30–54 years old	Needs-based
3	Ahern S et al., 2019	To develop a practical oral health needs based workforce planning simulation tool and apply it in a hypothetical situation using publicly available data in Ireland.	Ireland; WHO European Region; WB High Income Country	Dentists	Irish adults; 15+ years	Needs-based
4	Al-Jarallah KF et al., 2020	To describe the size of the dentist workforce in Kuwait between 1994 and 2006, and to project the future demand for dentists, and supply of Kuwaiti dentists for the years 2007–2020	Kuwait; WHO Eastern Mediterranean Region; WB High Income Country	Dentists	All population in Kuwait	Workforce to population ratio
5	Bourne CO, 2012	To estimate the current orthodontic manpower requirements of Trinidad and Tobago	Trinidad and Tobago; WHO American Region; WB High Income Country	Orthodontists	Children; 11 to 12 years	Workforce to population ratio
6	Brailsford S & De Silva D, 2015	To develop an operational model for informing policy decisions on number of dentists required in Sri Lanka	Sri Lanka; WHO South Asian Region; WB Lower Middle Income Country	Dentists	All population in Sri Lanka	Needs based demand weighted
7	Cao S et al., 2017	To determine the extent of paediatric dental care shortages in Georgia and to develop a general method for estimation that can be applied to other states	USA/Georgia; WHO American Region; WB High Income Country	Dentists; Paediatric dentists; Dental hygienists	Children aged under 18 years in Georgia, USA	Demand based
8	Cartes-Velásquez RA, 2013	To review the changes in academic, economic and workforce issues resulting from the growth in the supply of undergraduate dental vacancies between 1997 to 2011	Chile; WHO American Region; WB High Income Country	Dentists	All population in Chile	Workforce to population ratio
9	Eklund SA & Bailit HL, 2017	To examine factors that are linkely to affect the number of US dentists needed in 2040 and compare estimated number of dentists needed in 2040 to current trends.	USA; WHO American Region; WB High Income Country	Dentists	All population in USA	Demand based
10	Gallagher JE, Kleinman ER & Harper PR, 2010	To explore the required skill mix of the dental team to meet the future need and demand of older people in England.	UK/England; WHO European Region; WB High Income Country	Dentists; Hygienist; Therapist; Hygienist/Therapist; Clinical Dental Technicians	Older people (65 years)	Needs based, demand weighted
11	Gallagher JE, Lim Z & Harper PR, 2013	To explore future scenarios for the use of the skill mix within the dental team to inform the commissioning of dental therapy training.	UK/England/South Central SHA WHO European Region; WB High Income Country	Dentists; Dental therapists	All population based at South Central Strategic Health Authority (SHA), one of the 10 National Health Service (NHS) administrative zones in England.	Needs based, demand weighted
12	Gallagher JE, Manickam S & Wilson NHF, 2015	To describe trends in the dental workforce in Oman from 1990 to date; compare the dental workforce with its medical counterparts in Oman and with other countries; to consider future dental workforce in the Sultanate.	Oman; WHO Eastern Mediterranean Region; WB High Income Country	Dentists	All population in Oman	Workforce to population ratio
13	Huang CS et al., 2013	To make projections of the dental workforce from 2011 to 2020, based on a survey of the actual workload of 6762 dentists in 2010.	Taiwan; WHO Western Pacific Region; WB High Income Country	Dentists	All population in Taiwan	Workforce to population ratio
14	Ishimaru M et al., 2016	To estimate the future distribution of dentists with different working statuses in Japan and to discuss policy implications about the supply of dentists in any country.	Japan; WHO Western Pacific Region; WB High Income Country	Dentists	All population in Japan	Workforce to population ratio
15	Jaiswal AK et al., 2014	To analyse the changing trends in dental manpower production in India since 1920 and its development to date, including the number of dental colleges and distribution of trained professionals nationwide.	India; WHO South Asian Region; WB Upper Middle Income Country	Dentists	All population in India, broken by States/Territories.	Workforce to population ratio
16	Ju X et al., 2010	To estimate the supply and demand of oral and maxillofacial surgeons and services in Australia	Australia; WHO Western Pacific Region; WB High Income Country	Oral and Maxillo facial surgeons	Overall Australian population	Needs based, demand weighted
17	Mills RW, 2020	To gather data and help contribute towards assessing the need for future specialist training places by mapping General Dental Council (GDC)-listed specialists registered in UK postal areas and plotting specialists’ first GDC registration dates.	UK; WHO European Region; WB High Income Country	Dental Specialists	Not available (NA)	Workforce to population ratio
18	Saman DM, Arevalo O & Johnson AO, 2010	To assess geographic distribution of dentists in Kentucky; to estimate the future availability of dental providers and provide policy recommendations so as to improve access to oral health care in Kentucky and other rural states	USA/Kentucky; WHO American Region; WB High Income Country	Dentists	All population in Kentucky, USA	Workforce to population ratio
19	Shaw JL et al., 2017	To compare two methods of allocating general dentists to Canadian Armed Forces (CAF) dental detachments: a dentist-to-population ratio model and a needs-based model.	Canada; WHO American Region; WB High Income Country	Dentists	Canadian Armed Forces population in catchment areas	Workforce to population ratio
20	Sun X et al., 2017	To estimate the required human resources to meet the oral health needs of the WHO reference group of 12-year-olds in China and consider the implications for education, practice, policy and Human Resources for Oral Health nationally	China; WHO Western Pacific Region; WB Upper Middle Income Country	Dentists	Children; 12 year olds	Needs based
21	Surdu S et al., 2016	To evaluate the adequacy of the supply of paediatric dentists	USA; WHO American Region; WB High Income Country	Paediatric dentists	Children, all ages	Needs based demand weighted
22	Wanyonyi KL et al., 2015	To investigate the potential for skill mix use in primary dental care in England based on the undergraduate training experience in a primary care team training centre for dentists and mid-level dental providers	UK/England; WHO European Region; WB High Income Country	Dentists; Dental therapists	All population (adults and children) in England, who avail NHS public dental care.	Demand based
23	Zhang Y et al., 2015	To describe the distribution, structure and allocation of oral health services personnel, evaluate oral health service capacity and predict the future needs for oral health services in northern China.	China; WHO Western Pacific Region; WB Upper Middle Income Country	Dentists	All population based at Liaoning Province, China	Workforce to population ratio

**Table 2:** Detailed characteristics of supply and demand/needs model of selected studies.

Study No.	Author(s), Year	Supply Model		Demand|Needs Model			Modelling Technique
		Existing Stock; Flows; Newly trained; Workforce participation/Full Time Equivalent (FTE)	Data source(s)	Population; Demand|Needs Workforce requirement		Data source(s)	Approach Skill mix
1	Ab-Murat N et al., 2015	NA	NA	Population: 30–54 year old adults; employees at a public university in Kuala Lumpur, Malaysia (*n* = 732) Needs: Periodontal treatment needs assessed using Normative and Social Dental Approaches. - Normative Needs (NN) assessed using Community Periodontal Index, where presence of bleeding, calculus and pockets recorded for all indexed teeth. - Socio-Dental Need (SDA) assessed for people with NN. Impact Related Need and Propensity Related Need accounted for quality of life and behavioural assessment respectively in estimating treatment needs. Workforce requirement: Timings for periodontal procedures were used to estimate dental personnel required for both above approaches. Timings and dental personnel were estimated for 100,000 people to make inferences to all adults in Malaysia.		Oral health clinical examination of sample to assess oral health status and periodontal conditions. Face to face questionnaire survey to assess impacts on oral health related quality of life, frequency/severity of impacts, and oral health behaviours. Expert committee consisting of 6 dentists reviewed periodontal procedures and determined minimum and maximum times for periodontal treatments. The annual working hours of 1760 was used to highlight the differences in workforce estimates between the different scenarios	Approach: Dental personnel requirements for both NN and SDA estimated and compared using statistical tests. Comparisons made accounting for minimum and maximum treatment times, and dental personnel required for 100,000 Malaysian adults. Skill mix: Treatment timings for dentists and therapists were taken to be similar. Three scenarios modelled to meet periodontal care under the NN and SDA approaches: - Dentist only; - Minimum skill mix (only scaling and polishing delegated to therapists); - Maximum skill mix (scaling, polishing and root planning procedures delegated to dental therapists; dentists carry periodontal surgery)
2	Ab-Murat N et al., 2015	NA	NA	Population: 30–54 year old adults; employees at a public university in Kuala Lumpur, Malaysia (*n* = 732) Needs: Prosthodontic treatment needs assessed using Normative and Social Dental Approaches. - Normative Needs (NN) based on missing teeth, ill-fitting or non-aesthetic prosthesis. - Socio-Dental Need (SDA) assessed for people with NN. Impact Related Need measured via an oral impact for daily performance index. Propensity Related Need i.e., behavioural assessment for people who were in need of bridges or dentures Workforce requirement: Timings for prosthodontic treatment were used to estimate dental personnel required for both above approaches. Timings and dental personnel were estimated for 100,000 people to make inferences to all adults in Malaysia.		Oral health clinical examination of sample to assess oral health status including missing teeth, requirement of dentures or any prosthodontic treatment Face to face questionnaire survey to assess impacts on oral health related quality of life, frequency/severity of impacts, and oral health behaviours. Expert committee consisting of 6 dentists reviewed prosthodontic treatments for dentures and bridges, determined minimum and maximum times for periodontal treatments, 1760 annual working hours.	Approach: Dental personnel requirements for both NN and SDA estimated and compared using statistical tests. Comparisons made accounting for minimum and maximum treatment times, and dental personnel required for 100,000 Malaysian adults. Skill mix: Treatment timings for dentists and denturists were taken to be similar. Three scenarios modelled to meet prosthodontic care under the NN and SDA approaches: - Dentist only; - Minimum skill mix (denturists provide only complete dentures); - Maximum skill mix (denturists can provide all denture procedures except bridges)
3	Ahern S et al., 2019	Existing stock: Stock of dentists in Ireland was estimated using registration statistics, which included information such as date of registration, year of qualification and primary qualification. Flows: Inflows were based on three types: - Overseas-trained dentists newly registered to practice - Irish trained dentists returning to practice - Dentists returning after a period of absence Outflows were based on three types: - Dentists leaving Ireland - Dentists taking career break or period of absence - Retirement and death Newly trained: Number of undergraduate places in the two dental schools, adjusted for attrition/failure rate. Then, the number is adjusted to account for the percentage entering employment. Workforce participation/FTE: Participation rate assumed at 95% of all dentists registered, to account for dentists working in non-clinical activity. Further, activity rate was assumed to be at 85% accounting for part time work. All supply, inflow and outflow estimates were adjusted to reflect this activity rate.	Registration data of dentists from Dental Council of Ireland Registration data of dentists from Dental Council of Ireland Dental Schools Data	Population: Adult population of Ireland (15+ years). Age and gender distributions available. Needs: Oral health status mainly identified from four questions used in the population survey: - Number of teeth present - How often in past 12 months one has experienced difficulty in: - eating food due to oral problems - chewing/biting food due to oral problems - experienced toothache, mouth or denture problems Workforce requirement: Frequency and type of dental visits (check-up, routine or emergency) over past 12 months collected. Service timings (overall minutes and FTE dentists required) were estimated based on making assumptions on service timings.		Special Eurobarometer 330 Oral Health Survey dataset Special Eurobarometer 330 Oral Health Survey dataset Special Eurobarometer 330 Oral Health Survey dataset	Approach: Provider supply to requirement ratio estimated under four circumstances: - Unchanged current hours/times - Treatment times changed - Hours worked changed - Treatment times and hours worked both changed Projections available from 2017 to 2050. Skill mix: Not identified in the study/approach
4	Al-Jarallah KF et al. 2020	Existing stock Dentist estimates based on registrations data. Workforce participation/FTE: Dentist numbers (Kuwaiti and Non Kuwaiti) were used as proxy for workforce participation.	Dental Licensing Department; Ministry of Health Kuwait; Ministry of Planning Kuwait; Faculty of Dentistry, Kuwait	Population: Overall population of Kuwait was considered, including projections.		Ministry of Health Kuwait; Ministry of Planning Kuwait; Department of Statistics and Medical Records, Kuwait	Approach: Dentist supply numbers available from 1994 to 2006, and were projected for years 2007 to 2020. Similarly, population estimates were also available/projected for the given years. Projected supply estimates and dentist to population ratios accounted for growth in previous years. Shortfall in Kuwaiti dentists (based on annual increase estimates) to overall dentists (based on dentist to population ratio estimates) was projected for the years 2007 to 2020. Skill mix: Not identified in the study/approach
5	Bourne CO, 2012	NA	NA	Population: 11–12 year old childrenDemand: Orthodontic treatment preferences and visits of children were estimated through a population survey. Unclear how total children requiring orthodontic treatment were estimated. Workforce requirement: 9 orthodontists from 11 practices, who have been in practice for 10+ years and practice established 5 years ago were surveyed using a workforce questionnaire. Number of patients treated per year was estimated. Unclear how workforce requirements were limited to the specific age group 11–12 years.		Cross sectional study on orthodontic treatment needs of 11–12 years old children. Questionnaire survey administered to all orthodontists	Approach: Gap in total number of orthodontics required and currently available was identified though a survey based approach. Modelling aspects from the survey findings and how it applies to the specific age group is unclear in the description. A broad orthodontists to children/population approach seems to have applied for modelling. Skill mix: Not identified in the study/approach
6	Brailsford S & De Silva D, 2015	Existing stock: A national register of practising dentists natively prepared using dental registrations from medical council, record matching and panel interviews. Practice activity of dentists Flows: Attrition, Retirement and migration were accounted for in the model. Newly trained: Newly trained dentist practices based on outputs of the single dental school in Sri Lanka. Students were also surveyed on motivations and career expectations. Workforce participation/FTE: Practice activity of dentists surveyed via postal questionnaire, collecting information on socio-demographics, working patterns, hours worked, practice location, and main practice type. Total available clinical hours was estimated.	Sri Lankan Medical Council Registrations; Panel interviews; Dental practice activity survey Student survey	Population: All people in Sri Lanka Demand: FDI/WHO method for estimating services needed for a person, expressed in minutes, is used. Dental disease burden is accounted for in three main categories: caries, periodontal disease, and prosthodontic treatment needs. Local advice sought to identity percentage of people who need care, and who actively express demand for care. Workforce requirement: Timings for dental treatment were estimated via a survey, and treatment times were scaled up for each age groups, and at national level to identify the number of overall treatment hours required.		Sri Lankan Department of Census and Statistics 2003 National Oral Health Survey, Sri Lankan Ministry of Health	Approach: Supply and demands models separately created with the outputs of both models being number of hours available or required. Both these models were superimposed to identify gap in provision of services in treatment hours and number of dentists. A range of supply and demand scenarios were modelled for the years 2010 to 2024. Demand was considered in three main scenarios: low, moderate and high (in hours), and supply estimates were varied based on student intakes, retirements, practice activity restrictions, private sector participation and increased employment opportunities. Skill mix: Not identified in the study/approach
7	Cao S et al., 2017	Existing stock: Number of dental registrations available from local/state government sources. Flows: Not identified in the study/approach Newly trained: Not identified in the study/approach Workforce participation/FTE: Average work hours of 35.2 and 35.6 h accounted for male and female dentists per week. Time spend for the provision of paediatric dental care was estimated for both general dentists at 22% and paediatric dentists at 84%. This provided overall clinical hours available for treating children.	2015 National Plan and Provider Enumeration System Georgia Board of Dentistry roster for licenses expiring 2010 Survey of Dental Practice	Population: All children in Georgia, USA Demand: Caries risk estimated for children using survey data and prevalence of high risk and low risk children estimated across each census tract (geographic areas). Workforce requirement: Dental care demand per child by age group (0–3, 4–5, 6–7 and 8–18 years) estimated in minutes, and stratified by caries risk. Published data on procedure timings (including MEPS and expert opinions) were used to estimate paediatric treatment need. The timings were estimated for state and each geographic area/county.		2010 US Census data National Health and Nutrition Examination Survey data Medical Expenditure Panel Survey Expert opinion for treatment timings	Approach: Paediatric work hours were estimated both on supply side and demand side, and superimposed at the geographic level (country) to understand shortage areas. Skill mix: Dental hygienists, general dentists accounted for in the calculation of work hours along with paediatric dentists.
8	Cartes-Velásquez RA, 2013	Existing stock: Current workforce (baseline: 2012) estimated at *n* = 17,000 dentists. Historical data on number of dentists and graduates available from 1997 to 2011. Flows: Dentist migration and attrition rates were not included in the model. Newly trained: Current number of dental schools and graduates accounted for. Assumptions made that 80% of students in school graduate. Opening of new dental schools not accounted for. Workforce participation/FTE: Number of dentists available in the workforce was estimated based on the available assumptions at 2012, mainly accounting for student graduations. Unclear if any historical data from previous years were used to inform the supply projection model.	Indicadores de Instituciones y Carreras de Edu- cación Superior database of the Consejo Nacional de Educación (Education database from the Ministry of Education, Chile)	Population: Total population in Chile.		Instituto Nacional de Es- tadísticas de Chile (National Statistics Department of Chile)	Approach: Workforce to population ratios were estimated, and gap in dentist numbers and dentist to population ratios were visually described. Historical trends on dental school enrolments and dentist numbers also provided on the supply model.Skill mix: Not identified in the study/approach
9	Eklund SA & Bailit HL, 2017	Existing stock: Number of dentists (*n* = 195,722) in USA working across private practices, armed forces, hospitals, resident students or others were identified from a survey. Flows: Not identified in the study/approach Newly trained: Not identified in the study/approach Workforce participation/FTE: 70% of all dentists assumed to provide full time care (*n* = 136,905) at 30 or more hours per week.	American Dental Association Survey Centre	Population: Overall population for current year (2015) and projections for 2040 available. Demand: Previous publications suggest about 42 to 62% of people visit dentist once every year, estimated at 135 to 215 million single dental visits. Workforce requirement: Based on the number of dental visits, dentist requirements are estimated.		National Centre for Health Statistics Previous publications (Manski et al. 2009, 2016)	Approach: Number of FTE dentists available under the supply model and number of FTE dentists required under the demand model were estimated and compared. A range of theoretical assumptions and policy approaches were discussed, but without a modelling exercise. Skill mix: Not identified in the study/approach
10	Gallagher JE, Kleinman ER & Harper PR, 2010	Existing Stock: Supply of dentists, hygienists and therapists (2006: baseline) determined from registrations, survey and NHS data. The shift to dual qualified hygienists and therapists included, with a gradual increase in hygienist numbers. Flows: Short term recruitment drive (*n* = 1000 dentists) included; Unclear to what extent migration, attrition and return to work is incorporated. Newly trained: Current student completions, as well as increases in student intake for dentists (*n* = 170) and dental hygienists/therapists training (*n* = 150) accommodated. Workforce participation/FTE: Percentage of care provided for older people at NHS was estimated at 14% of all activity data. NHS FTE% for dentists was based on 69% of registrations, converting GDC register headcount to practising FTE dentists. NHS FTE for therapists/hygienists was assumed to be at 80% of NHS FTE of dentists.	Dental Practice Board of NHS England and Wales for old General Dental Services (GDS) and old Personal Dental Services (PDS); Primary Care Health Workforce Survey; General Dental Council Registrations	Population: Older people in England UK, aged 65 to 99 years. Demand: Population demographics (age, sex), oral health status (edentate rates), participation and attendance in NHS, and treatments provided included to estimate demand in terms of dentate and edentate treatments. Treatment rates, treatment times and costs were also accommodated for the services provided. Workforce requirement: Total demand was estimated in terms of treatment time (and cost)—also used in determining the FTE dentists and therapists/hygienists required.		UK Adult Dental Survey; The Information Centre UK; UK Government Actuary’s Department Treatment times from the British Dental Association	Approach: Workforce/FTE estimates were calculated using both the supply and demand models, and superimposition of both models identified shortage or surplus. Sensitivity analysis was done via Monte Carlo simulation and linear programming models. Estimates were projected from 2006 (baseline) to 2028. Skill mix: Various skill mix scenarios included that took into account the type of treatments provided by dentists and therapists/hygienists and FTE contribution (and costs). Five scenarios used: - Evolving skill mix - No skill mix - Hygenists/therapists expand tasks to include dental exams - Clinical Dental Technicians (CDTs) provide all dentures - Maximum skill mix, with expanded roles for hygienists/therapists and CDTs
11	Gallagher JE, Lim Z & Harper PR, 2013	Existing Stock: Dentist and dental therapist numbers were estimated for 1 NHS Administrative Zone: South Central Strategic Health Authority (SHA). Dentist numbers were available for baseline (2007) and past trend was accommodated for future years (2008 and 2013). Therapist numbers based on national dentist to therapist ratio (1:19). Flows: Migration, return to work and attrition were not identified in the model. Newly trained: New dental therapist training places (*n* = 34) accommodated for projection years 2008 and 2013. Workforce participation/FTE: NHS FTE considered for dentists and therapists; Final estimates are presented in workforce numbers i.e., number of dentists and therapists.	Information Centre UK; NHS South Central SHA; Local survey of skill mix from Buchinghamshire and Milton Keys Dental Workforce Survey	Population: All people at NHS South Central SHA Demand: Oral health trend, and proportion of treatment provided by NHS under 4 bands were available: - Band 1: Examination, diagnosis, preventive - Band 2: Band 1 + Routine treatment including fillings and extractions - Band 3: Band 1 + complex work such as dentures, crowns and bridges - Urgent/Emergency Proportion of care provided by therapists under each of these bands estimated. Three ages groups (0 to 19 years, 20–64 years and 65+ years) were used to estimate future dental demand. The model was developed with key parameters that affect the changes in needs and demand: demographic changes, oral health trends, dental attendance and proportion of treatments attended by each age group. Workforce requirement: NHS FTE potentially estimated to arrived at workforce numbers to meet demand.		The Information Centre UK; NHS South Central SHA	Approach: Supply and Demand models workforce outputs compared. Linear programming developed to obtain optimal makeup of workforce and project the future requirements of workforce supply. The model took various inputs estimates: treatments, cost/volume of activity, staff type or skill mix. Skill mix: A range of future scenarios were accounted in the models, which took into account use of therapists along with dentists.
12	Gallagher JE, Manickam S & Wilson NHF, 2015	Existing stock: Historical data from registrations/practice of dentists from 1990 to 2012 including stratification by Omani and Expatriate dentists identified. Projects from 2013 to 2020 estimated based on previous years. Flows: Migrant and Omani trained dentists accounted for in the estimates based on previous years. Migration and attrition maintained at constant levels for all years. Newly trained: Addition of new graduates to workforce form Oman Dental College maintained constant (*n* = 50) Workforce participation/FTE: Number of Oman and expatriate dentists	Ministry of Health Oman; World Health Organization	Population: Overall population of Oman and projections for years 1990 to 2020 available in the study. Population growth continues in an upward trajectory. 1990 to 2012 historical data. Projections 2013 onwards.		Ministry of Health, Oman	Approach: Dentist density or Dentist to population ratios calculated. Ratios—both for Omani dentists and Expatriate dentists. Three projection models were made for dentist to population ratios considered appropriate to meet: - WHO European benchmarks (1:2000) - Gulf Cooperative Council (GCC) benchmarks (1:3000) - Current global benchmarks (1:3800) Skill mix: Not identified in the study/approach
13	Huang CS et al., 2013	Existing stock: Number of total dentists (*n* = 11,449) and basic information on each dentist registered in Taiwan retrieved from the database of health personnel. Proportion of female dentists were also taken into account Flows: Attrition, retirement and migration were taken into account, and rate determined based on historical data. Newly trained: Yearly pass rates of dental licensing exam for 2006 to 2010 accounted for and average numbers included in the supply model. This pass rate also took into considerate number of foreign trained dentists. Workforce participation/FTE: Survey to dentists accounts for practice type and work hours, but clinical hours and FTE usage is unclear in the study. Workforce participation is represented as number of dentists available.	Department of Health database on health personnel, Executive Yuan (Taipei, Taiwan) Dentist practice activity survey	Population: All people in Taiwan. Both baseline and projections available from published government reports. Demand: A list of factors identified in the demand model including population change, increase in aged people, economic growth, new technology etc. But unclear on how they were used. Dentist to population ratios are presented as the means of comparing supply and demand		Official government reports of Council for Economic Planning and Development, Executive Yuan (Taipei, Taiwan)	Approach: Dentist to population ratios are estimated across 2010 to 2020. Supply and demand estimates are compared. Skill mix: Not identified in the study/approach
14	Ishimaru M et al., 2016	Existing stock: Number of dentists in Japan was estimated via a survey. Dentists registration number, year of registration, year of birth, sex, main working status, speciality and geographic postcodes of practice location were available from longitudinal survey data (1972 to 2012). Survey data is collected every 2 years. Flows: Retirees were identified as those who did not report to the for two consecutive surveys. Median age of retirees calculated at 65 years, and when dentists reach the retirement age they were considered as retirees. Newly trained: New entrants were identified via the matched cohort at 2012 from NSPDP. Workforce participation/FTE: Work status is identified as 6 main categories: ownership of practice; employed in dental clinic, hospital practice, academic work, and not reported. Workforce participation was represented via dentist numbers based on active registrations and work status.	National Survey of Physicians, Dentists and Pharmacists (NSPDP) for 1972–2012.	Population: Population numbers for Japan for years 1990 to 2012, and 2014 to 2042 used. Both historical estimates and population projections.		National Institute of Population and Social Security Research.	Approach: Dentists to population ratio (involving work status, and male/female dentists differentiators). Dentists work status across the six categories were identified for male and female dentists. Changes in distribution of work status accommodated for selected dentists at 0.5 and 10 years after registration for years 1982, 1992, 2002 and 2012. Probabilities for change in dentists work status were calculate for a wide range of patterns (78 patterns) for age and years of experience. Transition matrix models were developed using Markov chains to estimate future dentists. Skill mix: Not identified in the study/approach
15	Jaiswal AK et al., 2014	Existing stock: Based on dentists registrations across various states/territories. Number of dental institutions in India, undergraduate and postgraduate student positions obtained from the Dental Council of India. Flows: Not identified in the study Newly trained: Undergraduate placements in schools/colleges identified, but separately and not incorporated in the supply model. Workforce participation/FTE: Number of dentists used to identify workforce participation.	Central Bureau of Health Intelligence; Dental Council of India	Population: Population numbers for India, and individually for the States in India identified.	Ministry of Health and Family Welfare; Previous studies via literature		Approach: Dentist to population ratios were estimated using dentist registrations and population numbers, both for India, and individually for each of the States of India. This was determined for one year only (i.e., 2014). Growth in dental college numbers were provided from 1947 to 2014. Trends in increase in number of registered dentists from 1994 to 2012 was also provided separately, but not included as a main component of the modelling. Skill mix: Not identified in the study/approach
16	Ju X et al., 2010	Existing stock: Oral and maxillo facial surgeons (OMFS) estimates were available through from a survey, with information collected from speciality of practice question from a survey. OMFS estimates were available by age, sex and which state/territory they practised in Australia. Flows: Retirement, migration, cessation of practice or death accounted in the model. Attrition rates calculated based on male general dental practitioner wastage rates. Newly trained: Recruitment of OMFS was determined as the average number of completions between the years 2001 and 2005. This estimate was validated by the number trainees currently enrolled in OMFS training programs in Australia. Workforce participation/FTE: Participation was represented by the number of practising oral and maxillo facial surgeons; work status was obtained from the survey to determine practising oral and maxillo facial surgeons.	National Dental Labour Force Survey, 2006 Previous publication on all dentists in Australia for male general practitioner wastage rates; Source unclear for overseas entrants and returned to practice OMFS. Australasian Council of Dental Schools for recruitment vector;	Population: Population data for all Australians. Age specific information was included for 6 age groups. Demand: Six types of oral and maxillo facial services provided were identified: dentoalveolar, trauma, pathology, orthognathic, reconstructive surgery and other. Population estimates for people with oral and maxillo facial conditions were extrapolated based on service provision identified from the survey. Workforce requirement: Estimates on number of oral and maxillo facial surgeons required to meet the number of services estimated were calculated, and applied under different demand growth scenarios. It is unclear if FTE was used, or what is the ratio of surgeons to services utilised for demand.		Australian Bureau of Statistics for population data and projections Previous published reports on practice patterns and oral and maxillo facial services provided to patients.	Approach: Supply and demand models were developed separately and superimposed to identify gap in OMFS services. Both supply and demand projections were estimated from 2007 to 2037. Seven different types of supply scenarios and five different demand scenarios were used in the projection models. These models were later reconciled into three broad types: - Low supply and NO growth in demand - Medium supply and Half growth in demand - High supply and Continued growth in demand. Skill mix: Not identified in the study/approach
17	Mills RW, 2020	Existing stock: Number of dental specialists across all specialities in the UK were obtained from registrations data for 20 years (1999 to 2019). Postcode of practice location of these specialists were also obtained from the GDC registrations website information.	General Dental Council Specialist Registrations	NA		NA	Approach: Supply data of dental specialists were matched by postcode of practice location. Differences in practice location of specialities and lack of specialists in certain postcode areas were identified. Geographic information system approaches were used. Skill mix: Not identified in the study/approach
18	Saman DM, Arevalo O & Johnson AO, 2010	Existing stock: Dentist numbers were available from registrations, and location of practice mapped. Flows: Incoming and retiring dentists identified (change in results section). Newly trained: Not identified in the study/approach. Workforce participation/FTE: Number of dentists used for workforce participation.	Kentucky Board of Dentistry	Population: All population of Kentucky.		Kentucky State Data Centre	Approach: Dentist to population ratios were projected for each geographic area in Kentucky from 2007 up to 2016. The simulation model includes aspects of geospatial modelling to identify and map the dentist to population ratios. Skill mix: Not identified in the study/approach
19	Shaw JL et al., 2017	Existing stock: Number of dentists, specialists and allied dental practitioners available in the human resources management system. Flows: Not identified in the approach/study Newly trained: Not identified in the approach/study Workforce participation/FTE: FTE was calculated based on standard hours worked by a full time dentist (*n* = 1229.5 h). The number of clinical hours were adjusted to reflect clinical FTE, and variations in clinical provision adjusted across army ranks.	Human Resource Management System of Canadian Armed Forces (CAF) Canadian Forces Dental Services (CFDS) RESTORE & CFDS Position Charter (Policy documents)	Population: All people living in catchment areas served by the CAF clinics. Demand: Oral health status information of CAF personnel in catchment areas surveyed, along with treatment plan data. Workforce requirement: FTE requirement were estimated based on hours required to meet the demand.		Dental Information System (DentIS) Oral health surveillance data from a cross sectional sample of CAF personnel	Approach: FTE dentist requirements under both workforce to population ratio, and demand models were estimated. This calculation was extended to all geographic areas served under the CAF catchments. Level of FTE dentists agreement between the dentist to population model and demand model were assessed using Intraclass Correlation Coefficient and Bland-Altman plots. Skill mix: Though Skill mix in CAF is identified, only general dentists FTE seen in models presented.
20	Sun X et al., 2017	NA	NA	Population: Children; 12 year olds from 31 provinces of Mainland China, except Tibet. Needs: A representative sample of *n* = 23,508 children (12 years) clinically examined, and questionnaire survey for *n* = 12,392. Oral health status measured included dental caries experience and periodontal assessment. Oral health behaviours assessed via questionnaire. Four risk groups identified based on caries and behavioural assessment: - High risk - Low risk - Relatively high risk - Relatively low risk Workforce requirement: Risk based intervention models (maximum and minimum intervention) were developed and frequency of required dental visits estimated based on the four risk groups. Timings for care for each child were determined from panel of experts, and were aggregated to represent each child based on the risk level. Total timings for all 12 year olds in China was estimated using a population weighting approach. These timings for treatment were converted to workforce requirement based on average working hours per week (37.85 h per dental professional) to arrive at dental workforce numbers. Percentage of care provided for 12 year olds estimated at 1.27 percent. Similar workforce requirement was made for full population in China, based on 12 year olds.		3rd National Oral Health Survey Panel of nine experts from Peking University for data on timings for dental professionals Previous study (No 22) Wanyonyi et al., 2015 for data on percentage of care for 12 year olds.	Approach: Dental workforce requirements estimated based on a needs informed approach from a national population survey of 12 year olds in China. Percentage of time spent on 12 year olds was estimated, and workforce requirements both for 12 years olds and for all population in China was calculated. Skill mix: Not identified in the study/approach
21	Surdu S et al., 2016	Existing stock: Active paediatric dentists in the United States estimated using the membership data, and a 6% adjustment for non members. Overall *n* = 6530 paediatric dentists. Flows: Intended retirement age collected via online survey. All dentists were assumed to retire at 75 years. Age dependent attrition rates calculated either based on US mortality rates (for less than 50 years of age) or using survey responses. Annual cross state migration estimated via a logistic regression model using survey data on all dentists younger than 50 years. Unclear if overseas migration, and long term migration has been accounted for. Newly trained: Number of new graduates entering the paediatric dental workforce assumed based on previous publication/data (*n* = 448; 63.5% female). Age distribution of new graduates calculated based on new members data. Workforce participation/FTE: Information on patient care hours per week collected from an online survey of all paediatric dentists, who were association members with a US postal address. Ordinary least squares regression analysis used to model total weekly patient hours across various practice setting. FTE defined at 32.6 h per week in patient care activities.	American Association for Paediatric dentists membership data; Online survey of paediatric dentistry practice in 2016; Previous publication from American Dental Association (ADA), Health Policy Institute;	Population: Representative sample of child population for each state collected from US Census and Behavioural Risk Factor surveys (*n* = 656,400). This sample was weighted to represent the population in each state in the US (at national level summed up to 73.6 million children under 17 years of age). The population data contained information on age, sex, race, ethnicity, income, medical insurance and residence. Population projections 2015 to 2030 were made represent these sample characteristics, by scaling up the weights for the individual people in the sample. Demand: Patterns of care from MEPS survey were modelled to understand annual encounters to dental visits, considering age, sex, ethnicity, insurance, and geographic area as explanatory variables. Poisson regression was used to model patterns of annual care. All visits excluding prophylaxis and visits related to orthodontic procedures considered. Workforce requirement: Number of paediatric dentists required to meet supply were estimated to meet the demand, using various scenarios.		American Community Survey; Centre for Disease Control and Prevention 2014 and 2015 Behavioural Risk Factor Surveillance Data; US Mortality Register; Medical Expenditure Panel Survey (MEPS) 2010 to 2014 data.	Approach: Supply model based on a microsimulation approach to model future supply under a range of assumptions. Demand model for services based on patterns of care and visits using a range of population and oral health data. Sensitivity analysis was used for supply estimates; Weighting for both supply and demand estimates; least squares regression to model total weekly patient care hours; logistic regression to model interstate migration; Poisson regression to model annual care; Scenarios to model derived demand for dentists/paediatric dentists. Derived demand for dentists modelled using scenarios based on how workforce requirement will vary based on services and removal of barriers to access. Scenario 1: Continuation of care Scenario 2: Hypothetical—pediatric dentists provide care for all children under 4 years; 80% care for childre 5–12 years, and 20% care for children 13 to 17 years; - Scenario 3: Hypothetical; All children will have access to care and access barriers removed (approximates a needs-based scenario) - Scenario 4: Builds on Scenario 1 but including FTE estimates for general and paediatric dentists - Scenario 5: Builds on Scenario 3 but models FTE dentists. Kill mix: General and paediatric dentists modelled, but other skill mix considerations not identified.
22	Wanyonyi KL et al., 2015	Dentists and mid level providers (dental therapists) are identified as components of the overall model presented, but not accounted in the analysis or study results which focusses on alternative scenarios based on workforce requirements (based on demand but not compared with supply)	General Dental Council	Population: All population in England (both adults and children), who avail public dental care Demand: NHS dental services and treatment provided at a single site at South England were used to estimate age specific treatment rates across all NHS services for England. Timings for treatments were accounted using British Dental Association timings, and verified with expert panel. Total demand was expressed in age specific clinical hours. Workforce requirement: Workforce estimates were calculated based on the basis that dentists spend 0.4 FTE and hygienists/therapists spend 0.3 FTE of clinical hours for NHS work. Overall number of dentists and therapists required was expressed as alternative scenarios accounting for skill mix, and costs.		NHS Electronic Health Records Treatment data (single site at South England) British Dental Association timings for services Expert panel to validate treatment timings Salaries from National Career Service Advice	Approach: Treatment provision or utilisation data used in determining age specific treatment rates and timings of service. The later used to account for dentist/therapist FTE and costs based on skill mix alternative scenarios. Skill mix: Four NHS activity scenarios were used that accounted for delegation of tasks to dental hygienists/therapists: - Not skill mix - Minimal direct access - More prevention - Maximum delegation
23	Zhang Y et al., 2015	Existing stock: Survey of all practices in a single Province in China, including *n* = 2155 dental practices and *n* = 8611 oral health personnel (including dentists, nurses and technicians). Education, professional level, area of practice captured in the survey. Flows: Not identified in the study. Newly trained: Not identified in the study. Workforce participation/FTE: Hours worked collected in the survey, but its application towards the supply model is unclear.	Questionnaire survey sent to all dental facilities by Sanitation Bureau and Health Supervision station	Population: All population from a single Province (Liaoning) in China		Survey; Unclear	Approach: Number of dentists per population for the Province estimated (at current levels). Population projection for 2020 and workforce requirements to meet the population at 2020 was determined by using WHO recommended ratios. The gap in dental workforce for future identified, accounting a list of scenarios based on needs, amounts of time worked by dentists and dentist to population ratios Skill mix: Not identified in the study/approach

## Data Availability

All data presented in the study are available in the tables and figures included in this paper.

## Supplementary Materials

The following are available online at https://www.mdpi.com/1660-4601/18/6/2891/s1, Supplementary Table S1: Search Strategy for the Rapid Review; Supplementary Table S2: Limitations mentioned in selected studies. Supplementary Table S3: List of selected studies for Rapid Review with full citation. Supplementary Table S4: PubMed Search with MeSH terms.
