# Supplementary material for: Planning the Future Oral Health Workforce: A Rapid Review of Supply, Demand and Need Models, Data Sources and Skill Mix Considerations
Source: Int J Environ Res Public Health. 2021 Mar 12;18(6):2891. doi: 10.3390/ijerph18062891 (PMC7999471; doi:10.3390/ijerph18062891)
Supplement: Supplementary file 1 [file ijerph-18-02891-s001.pdf]

Supplementary Table 1: Search Strategy for the Rapid Review

PubMed (example)

(Healthcare workforce modelling in dentistry OR Healthcare workforce planning in dentistry OR Dental workforce planning OR Dental healthcare forecasting OR Health policy in dentistry OR Dental Manpower OR Dental Service Need* OR Dental workforce forecasting OR Dental workforce projections OR Dental workforce management OR Oral healthcare providers OR Human Resources for oral health OR Dental Projections OR Future dental workforce OR Dental workforce vision OR Dental workforce report OR Dental workforce review)
AND
(Dental staff levels OR Shortage of dentists OR Dental staff OR Dentists forecasting OR Dental hygiene therapists forecasting OR Dental Team OR Dental Manpower OR Manpower planning in dentistry OR Dental Professionals OR Oral Health Therapists)
AND
(Dentist-to-population ratio model OR Dental workforce model OR Dental Model* OR Modelling OR Workforce OR Demand models for healthcare workforce planning in dentistry OR Utilisation model in dentistry OR Needs based healthcare workforce planning OR Skill-mix OR Productivity OR Skill-mix in dentistry OR Dental activity and dental utilisation and workforce planning OR Workforce need OR Patterns of practice OR Meta ana$ OR Systematic review)

Supplementary Table 2: Limitations mentioned in selected studies

Study No.	Author(s), Year	Limitations
1	Ab-Murat N et al, 2015	- Single site study<br>- Adults (35-54 yrs) only<br>- Non working adults and rural population not included
2	Ab-Murat N et al, 2015	- Single site study<br>- Adults (35-54 yrs) only<br>- Non working adults and rural population not included
3	Ahern S et al, 2019	- Health status parameters (number of natural teeth and problem with food/pain) by gender and age remain constant throughout the planning period.<br>- Private health care not accounted in the model<br>- Shortcomings identified in availability of data.<br>-
4	Al-Jarallah KF et al, 2020	- Quality of data on dentist practice characteristics and dentist productivity were limited<br>- Dentist to population ratio has limitations as it holds constant the factors that affect population needs and desire for dental care.<br>- Uncertainty in projections have not been accounted for<br>- Data unavailable to study age structure of dentists and its effect on supply and demand.
5	Bourne CO, 2012	- Small sample size
6	Brailsford S & De Silva D, 2015	Not mentioned or Unclear
7	Cao S et al, 2017	- Did not account for differences in policies between Medicaid and private dental insurance<br>- Professional guidelines on type and frequency of services might not accurately represent demand<br>- Caries risk and time spent on preventive services obtained from national data, and might not reflect Georgia<br>- Dental procedure times were based on expert opinion<br>- Country boundaries defined for political reasons, and did not represent true dental markets<br>- No gold standard to determine how the model identified dental care shortage areas.<br>-
8	Cartes-Velásquez RA, 2013	- Incomplete records obtained on dental schools<br>- Lack of registration of all dental institutions to have data available<br>-
9	Eklund SA & Bailit HL, 2017	Not mentioned or Unclear
10	Gallagher JE, Kleinman	- General trade-off between model complexity to reflect system and simplicity so that its understandable<br>- Non availability of data

	ER & Harper PR, 2010	- Assumption that current level of demand is appropriate to need<br>- No consensus on what constitutes good care for older people in the future<br>- Older people care is not limited to a section of the workforce
11	Gallagher JE, Lim Z & Harper PR, 2013	- Quality of data from data sources<br>- Limited data on dental therapists both in numbers and practice activity
12	Gallagher JE, Manickam S & Wilson NHF, 2015	- Changes in population and dentist workforce assumptions influence dentist to population projections<br>- No use of skill mix
13	Huang CS et al, 2013	- Lack of availability in all supply and demand data
14	Ishimaru M et al, 2016	- Variations in dentist registrations and quality of data<br>- Assumptions made on supply and demand could change in the future
15	Jaiswal AK et al, 2014	Not mentioned or Unclear
16	Ju X et al, 2010	Not mentioned or Unclear
17	Mills RW, 2020	Not mentioned or Unclear
18	Saman DM, Arevalo O & Johnson AO, 2010	- Variations not necessarily descriptive of whole country<br>- Dentist to population ratio no a sufficient measure given there is no acceptable ratio<br>- Regression model has issues of multicollinearity<br>- Population migration not considered in the model
19	Shaw JL et al, 2017	- Treatment services data was broad, and could improve in granularity
20	Sun X et al, 2017	- Data sources used were not recent (oral health survey, census)<br>- Representativeness due to missing data in the survey<br>- Own methods used for caries risk assessment<br>- Reference timings for treatment from UK studies<br>- Lack of economic analysis
21	Surdu S et al, 2016	- Demand models did not take into account prevention.<br>- Skill mix not accommodated<br>- Uncertainty in key supply inputs due to limitations in data sources.
22	Wanyonyi KL et al, 2015	- Single site study<br>- Study was conducted in an educational establishment<br>- Lack of data on deprivation to estimate social parameters for need
23	Zhang Y et al, 2015	Not mentioned or Unclear

Supplementary Table 3: List of selected studies for Rapid Review with full citation

Study No	Citation
1	Ab-Murat N, Sheiham A, Tsakos G, Watt R. Periodontal treatment needs and workforce requirements: Comparisons between the normative and sociodental approaches using different skill mix models. Community Dent Oral Epidemiol. 2015;43(2):106–15.
2	Ab-Murat N, Sheiham A, Watt R, Tsakos G. Treatment needs and skill mix workforce requirements for prosthodontic care: a comparison of estimates using normative and sociodental approaches. BMC Oral Health [Internet]. 2015 Dec 13;15(1):36. Available from: http://bmcoralhealth.biomedcentral.com/articles/10.1186/s12903-015-0015-9
3	Ahern S, Woods N, Kalmus O, Birch S, Listl S. Needs-based planning for the oral health workforce - development and application of a simulation model. Hum Resour Health. 2019;17(5):1–9.
4	Al-Jarallah KF, Moussa MAA, Al-Duwairi Y, Zaatar E, Al-Khanfar KF. The dentist workforce in Kuwait to the year 2020. Community Dent Health. 2010;27(3):178–83.
5	Bourne CO. Orthodontic manpower requirements of Trinidad and Tobago. West Indian Med J. 2012;61(6).
6	Brailsford S, De Silva D. How many dentists does Sri Lanka need? Modelling to inform policy decisions. J Oper Res Soc. 2015;66(9):1566–77.
7	Cao S, Gentili M, Griffin PM, Griffin SO, Harati P, Johnson B, et al. Estimating demand for and supply of pediatric preventive dental care for children and identifying dental care shortage areas, Georgia, 2015. Public Health Rep. 2017;132(3):343–9.
8	Cartes-Velásquez RA. Exponential growth of dental schools in Chile: Effects on academic, economic and workforce issues. Braz Oral Res. 2013;27(6):471–7.
9	Eklund SA, Bailit HL. Estimating the Number of Dentists Needed in 2040. J Dent Educ. 2017;81(8):eS146–52.
10	Gallagher JE, Kleinman ER, Harper PR. Modelling workforce skill-mix: how can dental professionals meet the needs and demands of older people in England? Br Dent J [Internet]. 2010 Mar 13 [cited 2013 Oct 31];208(3):1–9. Available from: http://dx.doi.org/10.1038/sj.bdj.2010.106
11	Gallagher JE, Lim Z, Harper PR. Workforce skill mix: modelling the potential for dental therapists in state-funded primary dental care. Int Dent J [Internet]. 2013 Apr;63(2):57–64. Available from: http://doi.wiley.com/10.1111/idj.12006
12	Gallagher JE, Manickam S, Wilson NHF. Sultanate of Oman : building a dental workforce. Hum Resour Health [Internet]. 2015;13(50):1–10. Available from: http://dx.doi.org/10.1186/s12960-015-0037-z
13	Huang CS, Cher TL, Lin CP, Wu KM. Projection of the dental workforce from 2011 to 2020, based on the actual workload of 6762 dentists in 2010 in Taiwan. J Formos Med Assoc. 2013;112(9):527–36.
14	Ishimaru M, Ono S, Yasunaga H, Matsui H, Koike S. Projected future distribution of dentists in Japan. J Public Health Dent. 2016;76(3):241–8.
15	Jaiswal AK, Srinivas P, Suresh S. Dental manpower in India: changing trends since 1920. Int Dent J [Internet]. 2014;64:213–8. Available from: http://doi.wiley.com/10.1111/idj.12111
16	Ju X, Spencer A, Brennan D, ARCPOH (Australian Research Centre for Population Oral Health) The University of Adelaide. Supply and demand for oral and maxillofacial surgeons and services in Australia. Aust Dent J [Internet]. 2010;55(3):346–50. Available from: http://www.ncbi.nlm.nih.gov/pubmed/21166870
17	Mills RW. UK dental care for children - a specialist workforce analysis. Br Dent J. 2020;1–5.
18	Saman DM, Arevalo O, Johnson AO. The dental workforce in Kentucky: Current status and future needs. J Public Health Dent. 2010;70(3):188–96.

19	Shaw JL, Farmer JW, Coyte PC, Lawrence HP. Comparing human resource planning models in dentistry: A case study using Canadian Armed Forces dental clinics. Community Dent Oral Epidemiol. 2017;45(3):209–15.
20	Sun X, Bernabé E, Liu X, Zheng S, Gallagher JE. Meeting the oral health needs of 12-year-olds in China : human resources for oral health. BMC Public Health. 2017;17(586):1–12.
21	Surdu S, Dall TM, Langelier M, Forte GJ, Chakrabarti R, Reynolds RL. The pediatric dental workforce in 2016 and beyond. J Am Dent Assoc [Internet]. 2019;150(7):609-617.e5. Available from: https://doi.org/10.1016/j.adaj.2019.02.025
22	Wanyonyi KL, Radford DR, Harper PR, Gallagher JE. Alternative scenarios: Harnessing mid-level providers and evidence-based practice in primary dental care in England through operational research. Hum Resour Health. 2015;13(1):1–12.
23	Zhang Y, Lu Z, Cheng R, Liu L. Current state of allocation of oral health human resources in northern China and future needs. Int J Dent Hyg. 2015;13(4):268–72.

Supplementary Table 4: PubMed Search with MeSH terms

(((("delivery of health care"[MeSH Terms] OR ("delivery"[All Fields] AND "health"[All Fields] AND "care"[All Fields]) OR "delivery of health care"[All Fields] OR "healthcare"[All Fields] OR "healthcare s"[All Fields] OR "healthcares"[All Fields]) AND ("workforce"[MeSH Terms] OR "workforce"[All Fields] OR "workforces"[All Fields] OR "workforce s"[All Fields]) AND ("model"[All Fields] OR "model s"[All Fields] OR "modeled"[All Fields] OR "modeler"[All Fields] OR "modeler s"[All Fields] OR "modelers"[All Fields] OR "modeling"[All Fields] OR "modelings"[All Fields] OR "modelization"[All Fields] OR "modelizations"[All Fields] OR "modelize"[All Fields] OR "modelized"[All Fields] OR "modelled"[All Fields] OR "modeller"[All Fields] OR "modellers"[All Fields] OR "modelling"[All Fields] OR "modellings"[All Fields] OR "models"[All Fields]) AND ("dentistry"[MeSH Terms] OR "dentistry"[All Fields] OR "dentistry s"[All Fields])) OR (("delivery of health care"[MeSH Terms] OR ("delivery"[All Fields] AND "health"[All Fields] AND "care"[All Fields]) OR "delivery of health care"[All Fields] OR "healthcare"[All Fields] OR "healthcare s"[All Fields] OR "healthcares"[All Fields]) AND ("workforce"[MeSH Terms] OR "workforce"[All Fields] OR "workforces"[All Fields] OR "workforce s"[All Fields]) AND ("plan s"[All Fields] OR "planned"[All Fields] OR "planning"[All Fields] OR "plannings"[All Fields] OR "plans"[All Fields]) AND ("dentistry"[MeSH Terms] OR "dentistry"[All Fields] OR "dentistry s"[All Fields])) OR (("dental health services"[MeSH Terms] OR ("dental"[All Fields] AND "health"[All Fields] AND "services"[All Fields]) OR "dental health services"[All Fields] OR "dental"[All Fields] OR "dentally"[All Fields] OR "dentals"[All Fields]) AND ("workforce"[MeSH Terms] OR "workforce"[All Fields] OR "workforces"[All Fields] OR "workforce s"[All Fields]) AND ("plan s"[All Fields] OR "planned"[All Fields] OR "planning"[All Fields] OR "plannings"[All Fields] OR "plans"[All Fields])) OR (("dental health services"[MeSH Terms] OR ("dental"[All Fields] AND "health"[All Fields] AND "services"[All Fields]) OR "dental health services"[All Fields] OR "dental"[All Fields] OR "dentally"[All Fields] OR "dentals"[All Fields]) AND ("delivery of health care"[MeSH Terms] OR ("delivery"[All Fields] AND "health"[All Fields] AND "care"[All Fields]) OR "delivery of health care"[All Fields] OR "healthcare"[All Fields] OR "healthcare s"[All Fields] OR "healthcares"[All Fields]) AND ("forecasted"[All Fields] OR "forecaster"[All Fields] OR "forecasters"[All Fields] OR "forecasting"[MeSH Terms] OR "forecasting"[All Fields] OR "forecast"[All Fields] OR "forecasts"[All Fields] OR "trends"[MeSH Subheading] OR "trends"[All Fields])) OR (("health policy"[MeSH Terms] OR ("health"[All Fields] AND "policy"[All Fields]) OR "health policy"[All Fields]) AND ("dentistry"[MeSH Terms] OR "dentistry"[All Fields] OR "dentistry s"[All Fields])) OR (("dental health services"[MeSH Terms] OR ("dental"[All Fields] AND "health"[All Fields] AND "services"[All Fields]) OR "dental health services"[All Fields] OR "dental"[All Fields] OR "dentally"[All Fields] OR "dentals"[All Fields]) AND ("workforce"[MeSH Terms] OR "workforce"[All Fields] OR "manpower"[All Fields])) OR (("dental care"[MeSH Terms] OR ("dental"[All Fields] AND "care"[All Fields]) OR "dental care"[All Fields] OR ("dental"[All Fields] AND "service"[All Fields]) OR "dental service"[All Fields]) AND "need*"[All Fields]) OR (("dental health services"[MeSH Terms] OR ("dental"[All Fields] AND "health"[All Fields] AND "services"[All Fields]) OR "dental health services"[All Fields] OR "dental"[All Fields] OR "dentally"[All Fields] OR "dentals"[All Fields]) AND ("workforce"[MeSH Terms] OR "workforce"[All Fields] OR "workforces"[All Fields] OR "workforce s"[All Fields]) AND ("forecasted"[All Fields] OR "forecaster"[All Fields] OR "forecasters"[All Fields] OR "forecasting"[MeSH Terms] OR "forecasting"[All Fields] OR "forecast"[All Fields] OR "forecasts"[All Fields] OR "trends"[MeSH Subheading] OR "trends"[All Fields])) OR (("dental health services"[MeSH Terms] OR ("dental"[All Fields] AND "health"[All Fields] AND "services"[All Fields]) OR "dental health services"[All Fields] OR "dental"[All Fields] OR "dentally"[All Fields] OR "dentals"[All Fields]) AND ("workforce"[MeSH Terms] OR "workforce"[All Fields] OR "workforces"[All Fields] OR "workforce s"[All Fields]) AND ("forecasting"[MeSH Terms] OR "forecasting"[All Fields] OR "projected"[All Fields] OR "projecting"[All Fields] OR "projection"[MeSH Terms] OR "projection"[All Fields] OR "projections"[All Fields] OR "projectional"[All Fields])) OR (("dental health services"[MeSH Terms] OR ("dental"[All Fields] AND "health"[All Fields] AND "services"[All Fields]) OR "dental health services"[All Fields] OR "dental"[All Fields] OR "dentally"[All Fields] OR "dentals"[All Fields]) AND ("workforce"[MeSH Terms] OR "workforce"[All Fields] OR "workforces"[All Fields] OR "workforce s"[All Fields]) AND ("manage"[All Fields] OR "managed"[All Fields] OR "management s"[All Fields] OR "managements"[All Fields] OR "manager"[All Fields] OR "manager s"[All Fields] OR "managers"[All Fields] OR "manages"[All Fields] OR "managing"[All Fields] OR "managment"[All Fields] OR "organization and administration"[MeSH Terms] OR ("organization"[All Fields] AND "administration"[All Fields]) OR "organization and administration"[All Fields] OR "management"[All Fields] OR "disease management"[MeSH Terms] OR ("disease"[All Fields] AND "management"[All Fields]) OR "disease management"[All Fields])) OR (("mouth"[MeSH Terms] OR "mouth"[All Fields] OR "oral"[All Fields]) AND ("health personnel"[MeSH Terms] OR ("health"[All Fields] AND "personnel"[All Fields]) OR "health personnel"[All Fields] OR ("healthcare"[All Fields] AND "providers"[All Fields]) OR "healthcare providers"[All Fields])) OR (("workforce"[MeSH Terms] OR "workforce"[All Fields] OR ("human"[All Fields] AND "resources"[All Fields]) OR "human resources"[All Fields]) AND ("oral health"[MeSH Terms] OR ("oral"[All Fields] AND "health"[All Fields]) OR "oral health"[All Fields])) OR (("dental health services"[MeSH Terms] OR ("dental"[All Fields] AND "health"[All Fields] AND "services"[All Fields]) OR "dental health services"[All Fields] OR "dental"[All Fields] OR "dentally"[All Fields] OR "dentals"[All Fields]) AND ("forecasting"[MeSH Terms] OR "forecasting"[All Fields] OR "projected"[All Fields] OR "projecting"[All Fields] OR "projection"[MeSH Terms] OR "projection"[All Fields] OR "projections"[All Fields] OR "projectional"[All Fields])) OR (("forecasting"[MeSH Terms] OR "forecasting"[All Fields] OR "future"[All Fields] OR "futures"[All Fields]) AND ("dental health services"[MeSH Terms] OR ("dental"[All Fields] AND "health"[All Fields] AND "services"[All Fields]) OR "dental health services"[All Fields] OR "dental"[All Fields] OR "dentally"[All Fields] OR "dentals"[All Fields]) AND ("workforce"[MeSH Terms] OR "workforce"[All Fields] OR "workforces"[All Fields] OR "workforce s"[All Fields])) OR (("dental health services"[MeSH Terms] OR ("dental"[All Fields] AND "health"[All Fields] AND "services"[All Fields]) OR "dental health services"[All Fields] OR "dental"[All Fields] OR "dentally"[All Fields] OR "dentals"[All Fields]) AND ("workforce"[MeSH Terms] OR "workforce"[All Fields] OR "workforces"[All Fields] OR "workforce s"[All Fields]) AND ("vision s"[All Fields] OR "vision, ocular"[MeSH Terms] OR ("vision"[All Fields] AND "ocular"[All Fields]) OR "ocular vision"[All Fields] OR "vision"[All Fields] OR "visions"[All Fields] OR "visioning"[All Fields])) OR (("dental health services"[MeSH Terms] OR ("dental"[All Fields] AND "health"[All Fields] AND "services"[All Fields]) OR "dental health services"[All Fields] OR

"dental"[All Fields] OR "dentally"[All Fields] OR "dentals"[All Fields]) AND ("workforce"[MeSH Terms] OR "workforce"[All Fields] OR "workforces"[All Fields] OR "workforce s"[All Fields]) AND ("reportable"[All Fields] OR "reporting"[All Fields] OR "reportings"[All Fields] OR "research report"[MeSH Terms] OR ("research"[All Fields] AND "report"[All Fields]) OR "research report"[All Fields] OR "report"[All Fields] OR "reported"[All Fields] OR "reports"[All Fields])) OR (("dental health services"[MeSH Terms] OR ("dental"[All Fields] AND "health"[All Fields] AND "services"[All Fields]) OR "dental health services"[All Fields] OR "dental"[All Fields] OR "dentally"[All Fields] OR "dentals"[All Fields]) AND ("workforce"[MeSH Terms] OR "workforce"[All Fields] OR "workforces"[All Fields] OR "workforce s"[All Fields]) AND ("review"[Publication Type] OR "review literature as topic"[MeSH Terms] OR "review"[All Fields]))) AND ((("dental staff"[MeSH Terms] OR ("dental"[All Fields] AND "staff"[All Fields]) OR "dental staff"[All Fields]) AND ("level"[All Fields] OR "levels"[All Fields])) OR (("shortage"[All Fields] OR "shortages"[All Fields]) AND ("dentist s"[All Fields] OR "dentists"[MeSH Terms] OR "dentists"[All Fields] OR "dentist"[All Fields])) OR ("dental staff"[MeSH Terms] OR ("dental"[All Fields] AND "staff"[All Fields]) OR "dental staff"[All Fields]) OR (("dentist s"[All Fields] OR "dentists"[MeSH Terms] OR "dentists"[All Fields] OR "dentist"[All Fields]) AND ("forecasted"[All Fields] OR "forecaster"[All Fields] OR "forecasters"[All Fields] OR "forecasting"[MeSH Terms] OR "forecasting"[All Fields] OR "forecast"[All Fields] OR "forecasts"[All Fields] OR "trends"[MeSH Subheading] OR "trends"[All Fields])) OR (("oral hygiene"[MeSH Terms] OR ("oral"[All Fields] AND "hygiene"[All Fields]) OR "oral hygiene"[All Fields] OR ("dental"[All Fields] AND "hygiene"[All Fields]) OR "dental hygiene"[All Fields]) AND ("therapist"[All Fields] OR "therapist s"[All Fields] OR "therapists"[All Fields]) AND ("forecasted"[All Fields] OR "forecaster"[All Fields] OR "forecasters"[All Fields] OR "forecasting"[MeSH Terms] OR "forecasting"[All Fields] OR "forecast"[All Fields] OR "forecasts"[All Fields] OR "trends"[MeSH Subheading] OR "trends"[All Fields])) OR ("dent team"[Journal] OR ("dental"[All Fields] AND "team"[All Fields]) OR "dental team"[All Fields]) OR (("dental health services"[MeSH Terms] OR ("dental"[All Fields] AND "health"[All Fields] AND "services"[All Fields]) OR "dental health services"[All Fields] OR "dental"[All Fields] OR "dentally"[All Fields] OR "dentals"[All Fields]) AND ("workforce"[MeSH Terms] OR "workforce"[All Fields] OR "manpower"[All Fields])) OR (("workforce"[MeSH Terms] OR "workforce"[All Fields] OR "manpower"[All Fields]) AND ("plan s"[All Fields] OR "planned"[All Fields] OR "planning"[All Fields] OR "plannings"[All Fields] OR "plans"[All Fields]) AND ("dentistry"[MeSH Terms] OR "dentistry"[All Fields] OR "dentistry s"[All Fields])) OR (("dental health services"[MeSH Terms] OR ("dental"[All Fields] AND "health"[All Fields] AND "services"[All Fields]) OR "dental health services"[All Fields] OR "dental"[All Fields] OR "dentally"[All Fields] OR "dentals"[All Fields]) AND ("professional"[All Fields] OR "professional s"[All Fields] OR "professionalism"[MeSH Terms] OR "professionalism"[All Fields] OR "professionality"[All Fields] OR "professionalization"[All Fields] OR "professionalize"[All Fields] OR "professionalized"[All Fields] OR "professionalizing"[All Fields] OR "professionally"[All Fields] OR "professionals"[All Fields])) OR (("oral health"[MeSH Terms] OR ("oral"[All Fields] AND "health"[All Fields]) OR "oral health"[All Fields]) AND ("therapist"[All Fields] OR "therapist s"[All Fields] OR "therapists"[All Fields]))) AND (("Dentist-to-population"[All Fields] AND ("ratio"[All Fields] OR "ratio s"[All Fields] OR "ratioes"[All Fields] OR "ratios"[All Fields]) AND ("model"[All Fields] OR "model s"[All Fields] OR "modeled"[All Fields] OR "modeler"[All Fields] OR "modeler s"[All Fields] OR "modelers"[All Fields] OR "modeling"[All Fields] OR "modelings"[All Fields] OR "modelization"[All Fields] OR "modelizations"[All Fields] OR "modelize"[All Fields] OR "modelized"[All Fields] OR "modelled"[All Fields] OR "modeller"[All Fields] OR "modellers"[All Fields] OR "modelling"[All Fields] OR "modellings"[All Fields] OR "models"[All Fields])) OR (("dental health services"[MeSH Terms] OR ("dental"[All Fields] AND "health"[All Fields] AND "services"[All Fields]) OR "dental health services"[All Fields] OR "dental"[All Fields] OR "dentally"[All Fields] OR "dentals"[All Fields]) AND ("workforce"[MeSH Terms] OR "workforce"[All Fields] OR "workforces"[All Fields] OR "workforce s"[All Fields]) AND ("model"[All Fields] OR "model s"[All Fields] OR "modeled"[All Fields] OR "modeler"[All Fields] OR "modeler s"[All Fields] OR "modelers"[All Fields] OR "modeling"[All Fields] OR "modelings"[All Fields] OR "modelization"[All Fields] OR "modelizations"[All Fields] OR "modelize"[All Fields] OR "modelized"[All Fields] OR "modelled"[All Fields] OR "modeller"[All Fields] OR "modellers"[All Fields] OR "modelling"[All Fields] OR "modellings"[All Fields] OR "models"[All Fields])) OR (("dental health services"[MeSH Terms] OR ("dental"[All Fields] AND "health"[All Fields] AND "services"[All Fields]) OR "dental health services"[All Fields] OR "dental"[All Fields] OR "dentally"[All Fields] OR "dentals"[All Fields]) AND "model*"[All Fields]) OR ("model"[All Fields] OR "model s"[All Fields] OR "modeled"[All Fields] OR "modeler"[All Fields] OR "modeler s"[All Fields] OR "modelers"[All Fields] OR "modeling"[All Fields] OR "modelings"[All Fields] OR "modelization"[All Fields] OR "modelizations"[All Fields] OR "modelize"[All Fields] OR "modelized"[All Fields] OR "modelled"[All Fields] OR "modeller"[All Fields] OR "modellers"[All Fields] OR "modelling"[All Fields] OR "modellings"[All Fields] OR "models"[All Fields]) OR ("workforce"[MeSH Terms] OR "workforce"[All Fields] OR "workforces"[All Fields] OR "workforce s"[All Fields]) OR (("demand"[All Fields] OR "demanded"[All Fields] OR "demanding"[All Fields] OR "demands"[All Fields]) AND ("model"[All Fields] OR "model s"[All Fields] OR "modeled"[All Fields] OR "modeler"[All Fields] OR "modeler s"[All Fields] OR "modelers"[All Fields] OR "modeling"[All Fields] OR "modelings"[All Fields] OR "modelization"[All Fields] OR "modelizations"[All Fields] OR "modelize"[All Fields] OR "modelized"[All Fields] OR "modelled"[All Fields] OR "modeller"[All Fields] OR "modellers"[All Fields] OR "modelling"[All Fields] OR "modellings"[All Fields] OR "models"[All Fields]) AND ("delivery of health care"[MeSH Terms] OR ("delivery"[All Fields] AND "health"[All Fields] AND "care"[All Fields]) OR "delivery of health care"[All Fields] OR "healthcare"[All Fields] OR "healthcare s"[All Fields] OR "healthcares"[All Fields]) AND ("workforce"[MeSH Terms] OR "workforce"[All Fields] OR "workforces"[All Fields] OR "workforce s"[All Fields]) AND ("plan s"[All Fields] OR "planned"[All Fields] OR "planning"[All Fields] OR "plannings"[All Fields] OR "plans"[All Fields]) AND ("dentistry"[MeSH Terms] OR "dentistry"[All Fields] OR "dentistry s"[All Fields])) OR (("statistics and numerical data"[MeSH Subheading] OR ("statistics"[All Fields] AND "numerical"[All Fields] AND "data"[All Fields]) OR "statistics and numerical data"[All Fields] OR "utilization"[All Fields] OR "utilisation"[All Fields] OR "utilisations"[All Fields] OR "utilise"[All Fields] OR "utilised"[All Fields] OR "utilises"[All Fields] OR "utilising"[All Fields] OR "utilities"[All Fields] OR "utility"[All Fields] OR "utilizations"[All Fields] OR "utilize"[All Fields] OR "utilized"[All Fields] OR "utilizer"[All Fields] OR "utilizers"[All Fields] OR "utilizes"[All Fields] OR "utilizing"[All Fields]) AND ("model"[All Fields] OR "model s"[All Fields] OR "modeled"[All Fields] OR "modeler"[All Fields] OR "modeler s"[All Fields] OR "modelers"[All Fields] OR "modeling"[All Fields] OR "modelings"[All

Fields] OR "modelization"[All Fields] OR "modelizations"[All Fields] OR "modelize"[All Fields] OR "modelized"[All Fields] OR "modelled"[All Fields] OR "modeller"[All Fields] OR "modellers"[All Fields] OR "modelling"[All Fields] OR "modellings"[All Fields] OR "models"[All Fields]) AND ("dentistry"[MeSH Terms] OR "dentistry"[All Fields] OR "dentistry s"[All Fields])) OR (("health services needs and demand"[MeSH Terms] OR ("health"[All Fields] AND "services"[All Fields] AND "needs"[All Fields] AND "demand"[All Fields]) OR "health services needs and demand"[All Fields] OR "needed"[All Fields] OR "needs"[All Fields] OR "needing"[All Fields]) AND ("based"[All Fields] OR "basing"[All Fields]) AND ("delivery of health care"[MeSH Terms] OR ("delivery"[All Fields] AND "health"[All Fields] AND "care"[All Fields]) OR "delivery of health care"[All Fields] OR "healthcare"[All Fields] OR "healthcare s"[All Fields] OR "healthcares"[All Fields]) AND ("workforce"[MeSH Terms] OR "workforce"[All Fields] OR "workforces"[All Fields] OR "workforce s"[All Fields]) AND ("plan s"[All Fields] OR "planned"[All Fields] OR "planning"[All Fields] OR "plannings"[All Fields] OR "plans"[All Fields])) OR "Skill-mix"[All Fields] OR ("economics"[MeSH Terms] OR "economics"[All Fields] OR "production"[All Fields] OR "productions"[All Fields] OR "efficiency"[MeSH Terms] OR "efficiency"[All Fields] OR "productivity"[All Fields] OR "product"[All Fields] OR "product s"[All Fields] OR "productive"[All Fields] OR "productively"[All Fields] OR "productivities"[All Fields] OR "products"[All Fields]) OR ("Skill-mix"[All Fields] AND ("dentistry"[MeSH Terms] OR "dentistry"[All Fields] OR "dentistry s"[All Fields])) OR (("dental health services"[MeSH Terms] OR ("dental"[All Fields] AND "health"[All Fields] AND "services"[All Fields]) OR "dental health services"[All Fields] OR "dental"[All Fields] OR "dentally"[All Fields] OR "dentals"[All Fields]) AND ("activable"[All Fields] OR "activate"[All Fields] OR "activated"[All Fields] OR "activates"[All Fields] OR "activating"[All Fields] OR "activation"[All Fields] OR "activations"[All Fields] OR "activator"[All Fields] OR "activator s"[All Fields] OR "activators"[All Fields] OR "active"[All Fields] OR "actived"[All Fields] OR "actively"[All Fields] OR "actives"[All Fields] OR "activities"[All Fields] OR "activity s"[All Fields] OR "activitys"[All Fields] OR "motor activity"[MeSH Terms] OR ("motor"[All Fields] AND "activity"[All Fields]) OR "motor activity"[All Fields] OR "activity"[All Fields]) AND ("dental health services"[MeSH Terms] OR ("dental"[All Fields] AND "health"[All Fields] AND "services"[All Fields]) OR "dental health services"[All Fields] OR "dental"[All Fields] OR "dentally"[All Fields] OR "dentals"[All Fields]) AND ("statistics and numerical data"[MeSH Subheading] OR ("statistics"[All Fields] AND "numerical"[All Fields] AND "data"[All Fields]) OR "statistics and numerical data"[All Fields] OR "utilization"[All Fields] OR "utilisation"[All Fields] OR "utilisations"[All Fields] OR "utilise"[All Fields] OR "utilised"[All Fields] OR "utilises"[All Fields] OR "utilising"[All Fields] OR "utilities"[All Fields] OR "utility"[All Fields] OR "utilizations"[All Fields] OR "utilize"[All Fields] OR "utilized"[All Fields] OR "utilizer"[All Fields] OR "utilizers"[All Fields] OR "utilizes"[All Fields] OR "utilizing"[All Fields]) AND ("workforce"[MeSH Terms] OR "workforce"[All Fields] OR "workforces"[All Fields] OR "workforce s"[All Fields]) AND ("plan s"[All Fields] OR "planned"[All Fields] OR "planning"[All Fields] OR "plannings"[All Fields] OR "plans"[All Fields])) OR (("workforce"[MeSH Terms] OR "workforce"[All Fields] OR "workforces"[All Fields] OR "workforce s"[All Fields]) AND "need"[All Fields]) OR (("behavior"[MeSH Terms] OR "behavior"[All Fields] OR "behavioral"[All Fields] OR "behavioural"[All Fields] OR "behavior s"[All Fields] OR "behaviorally"[All Fields] OR "behaviour"[All Fields] OR "behaviourally"[All Fields] OR "behaviours"[All Fields] OR "behaviors"[All Fields] OR "pattern"[All Fields] OR "pattern s"[All Fields] OR "patternability"[All Fields] OR "patternable"[All Fields] OR "patterned"[All Fields] OR "patterning"[All Fields] OR "patternings"[All Fields] OR "patterns"[All Fields]) AND ("practicability"[All Fields] OR "practicable"[All Fields] OR "practical"[All Fields] OR "practicalities"[All Fields] OR "practicality"[All Fields] OR "practically"[All Fields] OR "practicals"[All Fields] OR "practice"[All Fields] OR "practice s"[All Fields] OR "practiced"[All Fields] OR "practices"[All Fields] OR "practicing"[All Fields])) OR (("meta"[Journal] OR "meta"[All Fields]) AND "ana"[All Fields]) OR ("systematic review"[Publication Type] OR "systematic reviews as topic"[MeSH Terms] OR "systematic review"[All Fields]))) AND ((english[Filter]) AND (2010:2020[pdat]))
